# Medical Society of Tennessee

**Published:** 1886-03-20

**Authors:** 


MEDICAL SOCIETY OF TENNESSEE—A circular, kindly sent by Dr. C. C. Fite, secretary, informs us that this Society will meet the present year in Memphis, on Tuesday, April 6, next. Dr. D. D. Sanders, of Memphis, is chairman of the committee of arrangements, and all communications in regard to the meeting should be addressed to him. Members who cannot be present can retain their membership and receive a copy of the Transactions by sending $1. Arrangements have been made to secure to the members return tickets at one-third rates. A large attendance is expected.

BARRY'S CLINICAL THERMOMETERS.—Among the best, if not the very best, clinical thermometers now in the market are those furnished by John Barry, manufacturer of Thermometers, at 62 Fulton street, New York. His thermometers come carefully and accurately tested and adjusted, and can be relied upon. We have one of these beautiful and excellent instruments, and find it in all respects satisfactory. This house also sells the Smith & Shaw closed cell Pocket Battery, which, for convenience, cheapness and efficiency, is highly recommended.

A NICE POCKET DRUG CASE.—A pocket drug case is a great convenience to the practitioner, and every practicing physician should carry one in his pocket continually. It is well to select one which is large enough to carry a sufficient variety of drugs, and yet not too large for the pocket. One of the best and most beautiful drug cases we have seen is that presented as a prize in Physiology at the late commencement exercises of the Southern Medical College of this city. It was from that splendid Baltimore drug house of Sharp, Dohme & Co., and contained twenty-four 2-drachm phials, loaded with beautiful granules and sugar-coated pills, so well and neatly prepared by that establishment. Those desiring to buy should address the firm at Baltimore, Md. See their advertisement on second page of cover.

